# Research Hotspots and Emerging Trends of Schizophrenia and Immune Response: A Bibliometric Analysis

**DOI:** 10.1002/brb3.71054

**Published:** 2025-11-21

**Authors:** Yu Zhang

**Affiliations:** ^1^ Department of Psychiatry, the Fourth Affiliated Hospital of School of Medicine, and International School of Medicine, International Institutes of Medicine Zhejiang University Yiwu China

**Keywords:** immune response, neuroinflammation, oxidative stress, schizophrenia

## Abstract

**Introduction:**

Schizophrenia is a complex psychiatric disorder increasingly recognized for its association with immune responses. This bibliometric analysis aims to systematically evaluate global research trends, emerging themes, and collaborative networks in the study of schizophrenia and immune response.

**Methods:**

Publications related to schizophrenia and immune response were retrieved from the Web of Science Core Collection. Bibliometric tools, including VOSviewer, CiteSpace, and the R package “bibliometrix,” were used to analyze keyword co‐occurrence and collaboration networks among countries, institutions, journals, and authors.

**Results:**

A total of 1556 articles were published between 1980 and 2024, with an annual growth rate of 11.39%. The United States led globally in both publication volume (364 articles) and citation count (18,328 citations). The University of California system and Harvard University were the leading institutions, whereas *Brain, Behavior, and Immunity* was the most influential journal. Maes M. ranked first in publication output with 35 articles. Co‐occurrence analysis of keywords identified five research clusters: neuroinflammation and cellular mechanisms, immune markers and clinical responses, autoimmunity and peripheral immune systems, genetic susceptibility and molecular pathways, and neuroinflammation and stress responses. The keyword “disorder” experienced a sustained citation burst in 2024.

**Conclusion:**

This study indicates that research on schizophrenia and immune responses remains focused on mechanistic exploration. Future studies should prioritize integrating clinical manifestations with molecular mechanisms, combining multi‐omics data with clinical phenotypes to deeply investigate the role of immune regulation in disease pathogenesis and treatment, thereby advancing precision medicine.

## Graphical abstract

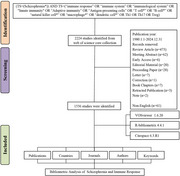

## 1 Introduction

Schizophrenia is a severe mental disorder characterized by multifaceted impairments in cognition, perception, emotion, and behavior (Jauhar et al. 2022). Its core pathological features include hallucinations, delusions, disorganized thinking, emotional blunting, reduced volition, social withdrawal, and cognitive deficits (e.g., in attention, memory, and executive function) (de Winter et al. 2025). Epidemiological studies indicate a lifetime prevalence of 0.7–1, with higher incidence in males (Li et al. 2022). Its complex pathogenesis involves interactions among genetic, neurodevelopmental, and environmental factors (Jauhar et al. 2022; Toriumi et al. 2025; Owen et al. 2023).

Immune response, the biological defense mechanism of the body against pathogens or abnormal cells, involves both innate and adaptive immunity (Wang et al. 2024). Dysregulation of the immune system has been linked to schizophrenia through mechanisms such as chronic low‐grade inflammation, cytokine imbalances, and autoimmune responses that affect the central nervous system (Yan et al. 2023). For instance, maternal infections during pregnancy, antibody positivity to pathogens such as *Toxoplasma gondii*, and gut microbiome dysbiosis have been associated with an increased risk of schizophrenia (Lins 2021). Immune‐related biomarkers, such as elevated C‐reactive protein (CRP) levels, have been correlated with disease severity and therapeutic responses in schizophrenia (Balcioglu and Kirlioglu 2020). Furthermore, clinical trials investigating immunomodulatory therapies, including adjunctive anti‐inflammatory drugs, have shown promise in alleviating negative symptoms of the disorder (Chandra et al. 2023). These findings underscore the growing recognition of immune system involvement in schizophrenia, offering a fresh perspective on its pathophysiology beyond traditional neurotransmitter‐based hypotheses. Research in this field has increasingly adopted advanced methodologies, such as multi‐omics approaches, to integrate clinical phenotypes with molecular data and elucidate immune‐mediated heterogeneity in schizophrenia (Guan et al. 2021). This evolving framework not only deepens our understanding of the immune microenvironment's role in the disorder and provides insights that could inform the development of targeted interventions and biomarkers in future research.

Despite the growing body of literature on the interplay between immune response and schizophrenia, previous reviews and meta‐analyses have largely focused on specific biological mechanisms, genetic associations, or therapeutic interventions (van Kesteren et al. 2017; Ermakov et al. 2022). Although bibliometric analyses in related fields, such as psychoneuroimmunology and schizophrenia biomarkers, have been conducted, these studies often target narrower aspects of the research landscape or focus on specific subtopics. For example, prior bibliometric reviews have concentrated on psychoneuroimmunology's broader relevance to psychiatric disorders or the role of specific biomarkers in schizophrenia. However, none have provided a comprehensive mapping of research trends specifically combining schizophrenia and immune response.

Bibliometric analysis, a statistical method for evaluating research trends and collaborations, provides several advantages over traditional reviews or meta‐analyses. Although meta‐analyses synthesize findings from selected studies to answer specific clinical or mechanistic questions, bibliometrics offers a broad, quantitative overview of the entire research field by mapping publication outputs, citation networks, and intellectual structure across decades (Chen et al. 2020). This enables the identification of influential contributors, journals, and collaborative networks, as well as the detection of research hotspots and gaps at a macro level.

In the context of schizophrenia and immune response, this study uniquely integrates bibliometric methods to systematically analyze interdisciplinary patterns, global collaborations, and thematic shifts over time. By focusing on the intersection of schizophrenia and immune mechanisms rather than general psychoneuroimmunology or schizophrenia biomarkers, this work provides novel insights into emerging areas such as neuroinflammation, immune markers, and multi‐omics approaches. Furthermore, this study aims to highlight underexplored research directions while offering a comparative evaluation of global contributions.

Therefore, the aim of this study is to provide a systematic, data‐driven mapping of the global research landscape on schizophrenia and immune response. Specifically, we seek to (1) identify major trends and patterns in research output, (2) determine the most influential countries, institutions, and authors, and (3) uncover key research clusters and emerging themes through keyword and collaboration analyses.

## 2 Materials and Methods

### 2.1 Search Strategies and Data Collection

A comprehensive bibliometric analysis was performed using the Web of Science Core Collection (WoSCC), a leading database known for its coverage of authoritative scholarly literature across various disciplines (Liu et al. 2024). The search strategy employed the following query: (TS = (schizophrenia*)) AND TS = (“immune response” OR “immune system” OR “immunological system” OR “innate immunity” OR “adaptive immunity” OR “antigen‐presenting cells” OR “T cell*” OR “B cell*” OR “natural killer cell*” OR “macrophage*” OR “dendritic cell*” OR Th1 OR Th17 OR Treg) (Sun et al. 2022; Xing et al. 2024). The search was restricted to original research articles published in English. The inclusion criteria were as follows: (1) original research articles that focused on schizophrenia and immune response, as defined by the presence of relevant keywords in the title, abstract, or keywords; (2) articles published in peer‐reviewed journals; and (3) articles published in English. The exclusion criteria were (1) review articles, conference proceedings, editorials, letters, and book chapters; (2) articles not directly related to both schizophrenia and immune response; and (3) duplicate records.

All data retrieval was conducted on January 15, 2025. A rigorous, two‐step screening process was then independently performed by two authors to ensure relevance and rigor. These authors first screened all titles and abstracts and then proceeded to independent full‐text reviews of potentially eligible articles to confirm relevance against the inclusion/exclusion criteria. Discrepancies at any stage were resolved through discussion to reach consensus or by consulting a third reviewer. The data were exported using the “full record and cited references” and “plain text” formats, which provided comprehensive information, including publication metrics, citation data, author details, institutional affiliations, geographic distributions, keywords, and journal indicators. Despite the broad search strategy, this multi‐step screening process minimized the risk of including irrelevant articles.

### 2.2 Statistical Analysis

The data analysis involved three primary bibliometric tools: VOSviewer (version 1.6.20), CiteSpace (version 6.3.R1), and the R package “bibliometrix” (version 4.4.3). VOSviewer was employed to visualize scientific networks, including countries, institutions, authors, and keyword co‐occurrence networks (Van Eck and Waltman 2010). Node sizes represented the relative importance of an entity (e.g., author, institution). Line thickness represented the strength of collaboration or co‐occurrence, whereas color schemes were applied to represent different temporal periods or cluster memberships, providing further insight into the evolution of research themes. For CiteSpace, parameters were set with time slicing at 1‐year intervals, the selection of the top five most cited items per slice, and the use of pathfinder network scaling to prune the network. This configuration facilitated the detection of emerging trends and temporal shifts in the research landscape (Li et al. 2024). To assess the scientific impact, the R package “bibliometrix” was extensively used to conduct statistical analysis and advanced bibliometric computations.

Key bibliometric indicators, such as publication counts, *H*‐index, *G*‐index, and *M*‐index, were evaluated to assess the academic influence of authors, journals, and institutions (Bertoli‐Barsotti and Lando 2017; Hirsch 2005). Journal impact metrics, including Journal Citation Reports (JCR) quartiles and impact factors (IF), were extracted to determine journal prestige and citation influence. JCR quartiles categorize journals into four levels, with Q1 representing the highest tier of academic quality, whereas the IF indicates the average citation rate over the preceding 2 years (Hirsch 2005).

## 3 Results

### 3.1 Publication and Citation Trends Analysis

**FIGURE 1:**
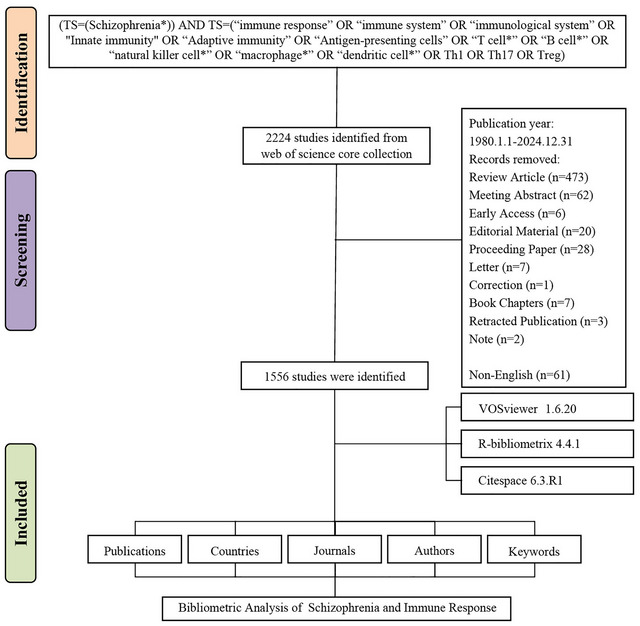
Flow diagram of the bibliographic retrieval process, illustrating each step of the study selection and inclusion criteria.

From 1980 to 2024, research exploring the relationship between schizophrenia and immune response has shown remarkable growth, with 1556 documents published during this period and an annual growth rate of 11.39% (Figure 1). These publications involved 8912 authors, of which only 38 authored single‐authored documents. International collaboration was significant, with 35.22% of the publications involving international co‐authorship, and the average number of co‐authors per document was 8.12.

**FIGURE 2:**
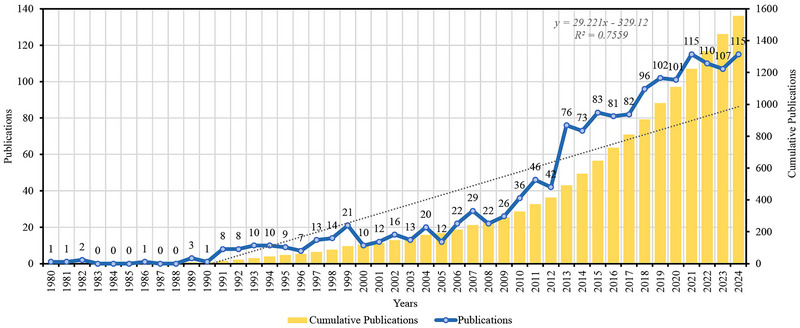
Annual output of schizophrenia and immune response research from 1980 to 2024, demonstrating the steady increase in publications and the recent surge in interest.

The publication trend reveals three distinct phases of growth (Figure 2). The initial phase (1980–1999) showed modest publication activity with fewer than 20 papers annually. A transitional phase occurred between 2000 and 2010, with publications steadily increasing to around 40–50 papers per year. The field experienced rapid expansion from 2011 onwards, reaching over 100 publications annually by 2019 and peaking at 2021 and 2024 with 115 publications.

### 3.2 Analysis of Leading Countries

**FIGURE 3:**
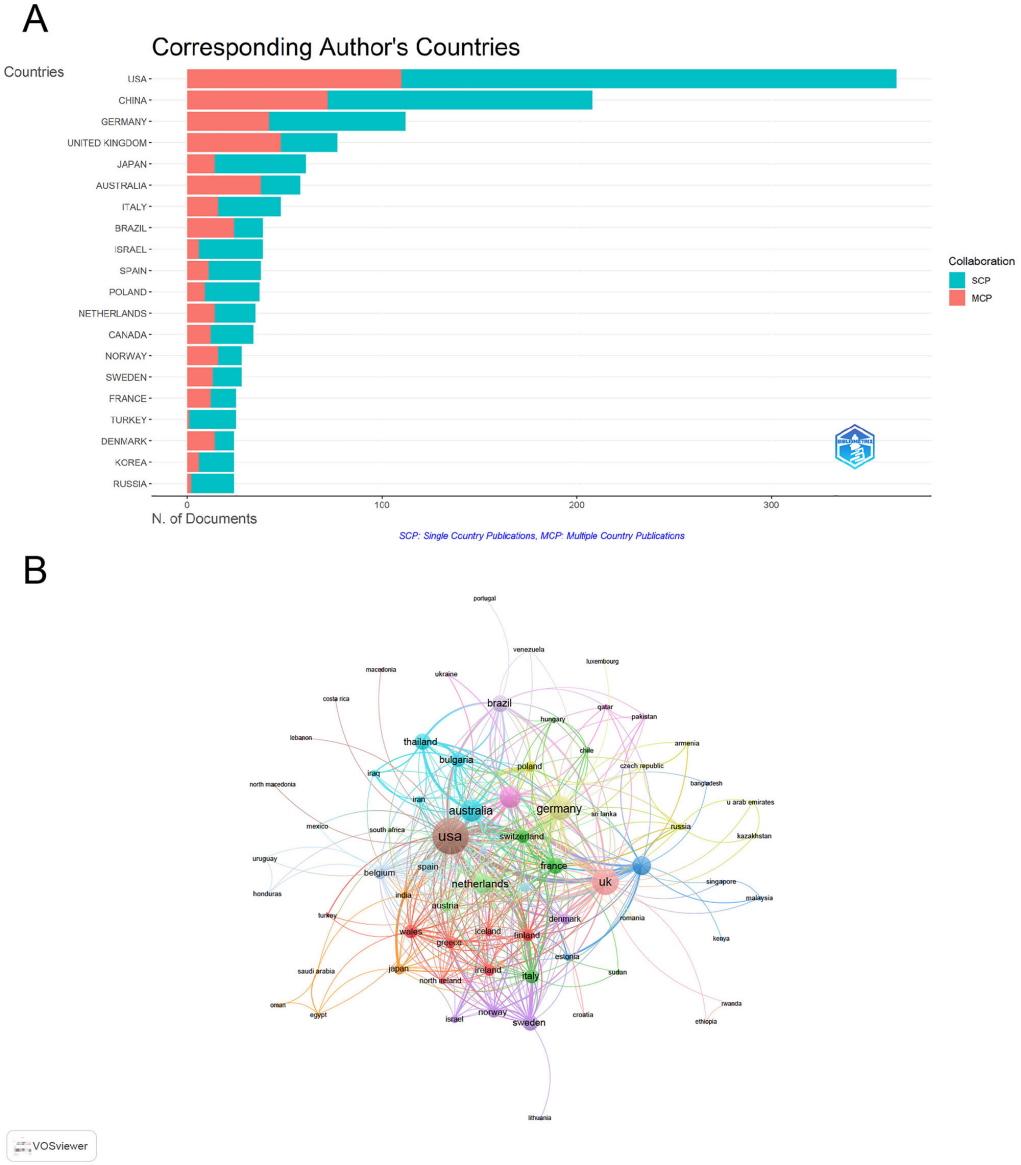
Analysis of countries contributing to schizophrenia and immune response research. (A) Distribution of corresponding author publications by country, highlighting the leading contributors (United States, China, and Germany). (B) Visualization map depicting international collaboration patterns among countries, emphasizing strong partnerships and areas for improvement in global research networks.

The global distribution of publications revealed significant contributions from multiple countries, with distinct patterns of collaboration and impact. The United States led with 364 articles (23.4%), followed by China with 208 articles (13.4%), and Germany with 112 articles (7.2%). These findings highlight the dominant role of the United States, which not only leads in publication volume but also in influence, as evidenced by its total of 18,328 citations and an average of 50.4 citations per document. This reflects the country's strong research infrastructure and funding in areas of psychiatry and immunology. Notably, the United Kingdom ranked second in total citations (TC) (8572) and achieved the highest average citations per document at 111.3. Germany followed in third place with 5706 citations and an average of 50.9 citations per document. The United States demonstrated the highest multiple‐country publication (MCP) number of 110, followed by China (136) and Germany (70) (Figure 3A and Table S1). The high MCP values underline the importance of international collaboration in advancing research on schizophrenia and immune response. For example, the United States’ strong collaborative network (total link strength = 487) with countries such as the United Kingdom (268) and Germany (215) suggests that cross‐border partnerships are central to generating impactful research (Figure 3B). This further indicates the global nature of this interdisciplinary field, where collaborative efforts are needed to address complex research questions.

### 3.3 Analysis of Institutions

**FIGURE 4:**
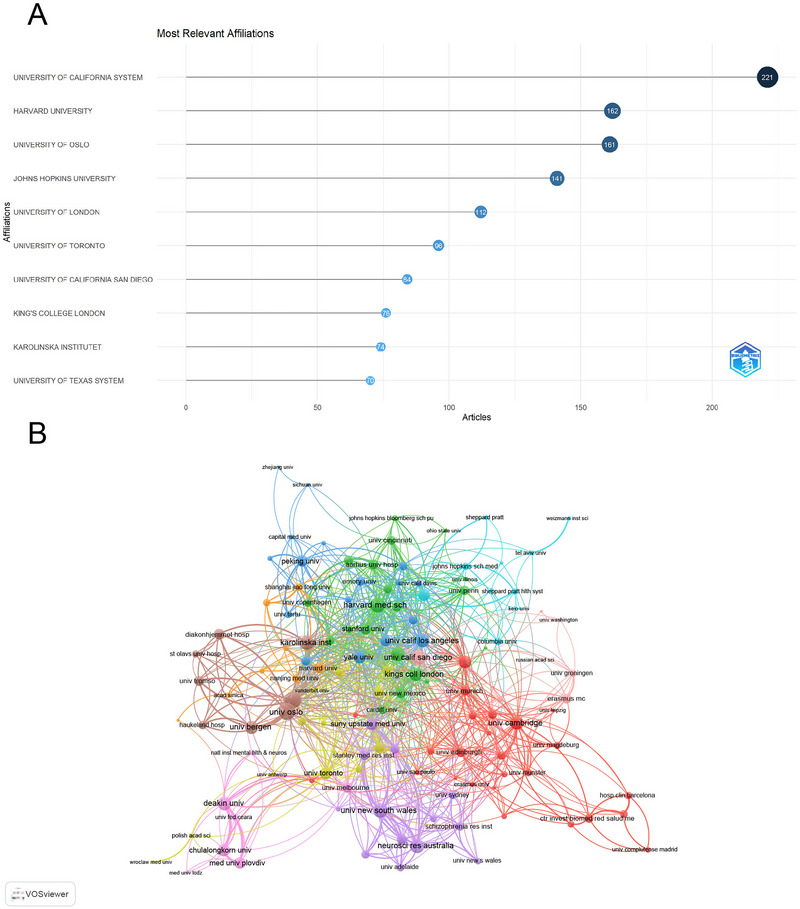
Analysis of institutions contributing to schizophrenia and immune response research. (A) Top 10 institutions ranked by article count, illustrating the dominance of institutions like the University of California System and Harvard University. (B) Visualization map depicting collaboration among institutions, showing the strength of interdisciplinary and inter‐institutional research efforts.

Institutional analysis revealed the University of California System as the leading contributor with 221 publications, followed by Harvard University (162) and the University of Oslo (161) (Figure 4A). The prominence of the University of California System and Harvard University reflects the robust contributions of US‐based institutions, which benefit from substantial funding and well‐established research programs in psychiatry and immunology. The institutional collaboration network demonstrated (Figure 4B) that among the 129 institutions with a minimum of 7 articles, the University of Oslo had the highest number of collaborations with other countries (total link strength = 116), followed by Oslo University Hospital (111) and Harvard University (89). The high collaboration strength of the University of Oslo and Oslo University Hospital reflects the growing role of European institutions in fostering international partnerships. Such collaborations are vital for leveraging diverse expertise and resources to tackle the complex interplay between schizophrenia and immune mechanisms.

### 3.4 Analysis of Authors

The analysis of author contributions revealed significant variations in research productivity and impact ranked by *H*‐index (Table S2). Maes M. emerged as the most prolific author with 35 publications and an *H*‐index of 21, followed by Müller N. (21, 19) and Yolken R. H. (27, 19). Maes M.’s prolific output and high *H*‐index suggest a central role in advancing the understanding of immune dysfunction in schizophrenia. Similarly, Yolken R. H.’s high *M*‐index (1.188) reflects sustained productivity and influence in this area, particularly in studies linking infectious agents and immune markers to schizophrenia. These findings underscore the contributions of key researchers who have shaped the field's focus on integrating immunological insights with psychiatric research.

**FIGURE 5:**
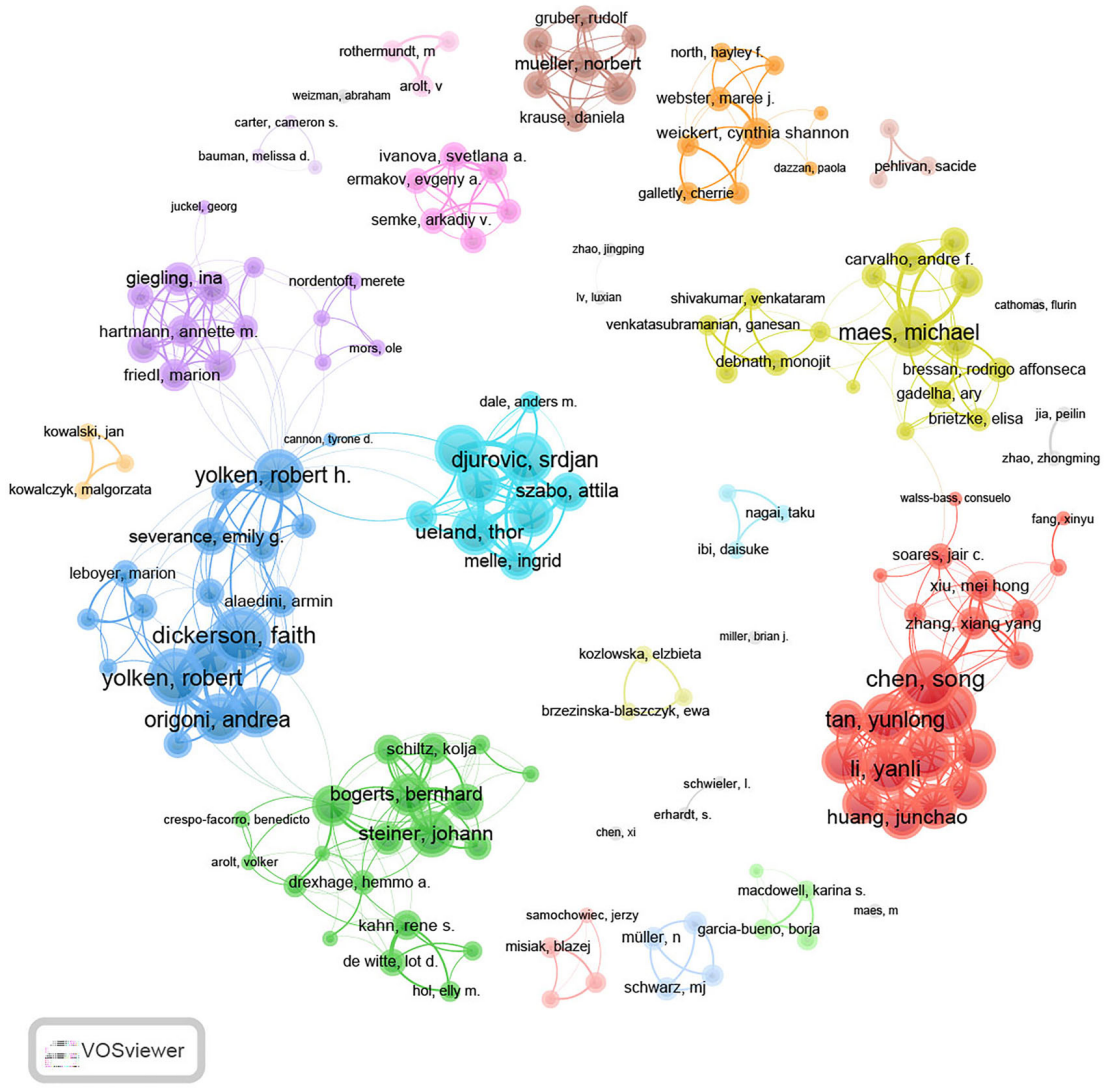
Visualization map depicting collaboration among authors, identifying key researchers and their collaborative networks.

Author collaboration networks (Figure 5) include authors with a minimum of five publications and reveal several key clusters. For example, the red cluster predominantly includes authors from China, such as Chen Song, Tan Yunlong, and Huang Junchao. In contrast, the blue cluster, centered around international researchers like Yolken Robert and Dickerson Faith, shows a more globally connected pattern, with extensive collaborations across different countries. Similarly, the yellow cluster, led by Maes Michael and Carvalho Andre F., reflects strong links among European researchers. These distinct clusters highlight regional and international collaboration patterns, with certain researchers acting as hubs for cross‐disciplinary partnerships. For instance, the blue cluster's global connectivity reflects the importance of international integration in generating impactful studies, whereas the red cluster emphasizes the growing contributions of Chinese researchers in this field.

### 3.5 Analysis of Journals

The journal analysis revealed that *Brain, Behavior, and Immunity* led with the highest *H*‐index (37) and IF (8.8, 2023), as well as the highest total publications (TP = 96) among journals in this field. This journal also ranked fourth in TC (=1877). *Schizophrenia Research* (IF = 3.6) followed with an *H*‐index of 36 and TP of 84 (ranked second), making it the most cited journal overall (TC = 3253). *Schizophrenia Bulletin* (IF = 5.3) ranked third in *H*‐index (25) and fifth in TC (=1832), with TP of 38 (ranked sixth) (Table S3). These findings illustrate that *Brain, Behavior, and Immunity* has become a central platform for publishing research at the intersection of psychiatry and immunology, reflecting its ability to attract high‐quality interdisciplinary studies. *Schizophrenia Research*, on the other hand, remains a cornerstone for schizophrenia‐specific studies, with its high TC and influence in shaping the field. The prominence of *Schizophrenia Bulletin* highlights its role in advancing in‐depth understanding of schizophrenia's pathophysiology and clinical implications.

**FIGURE 6:**
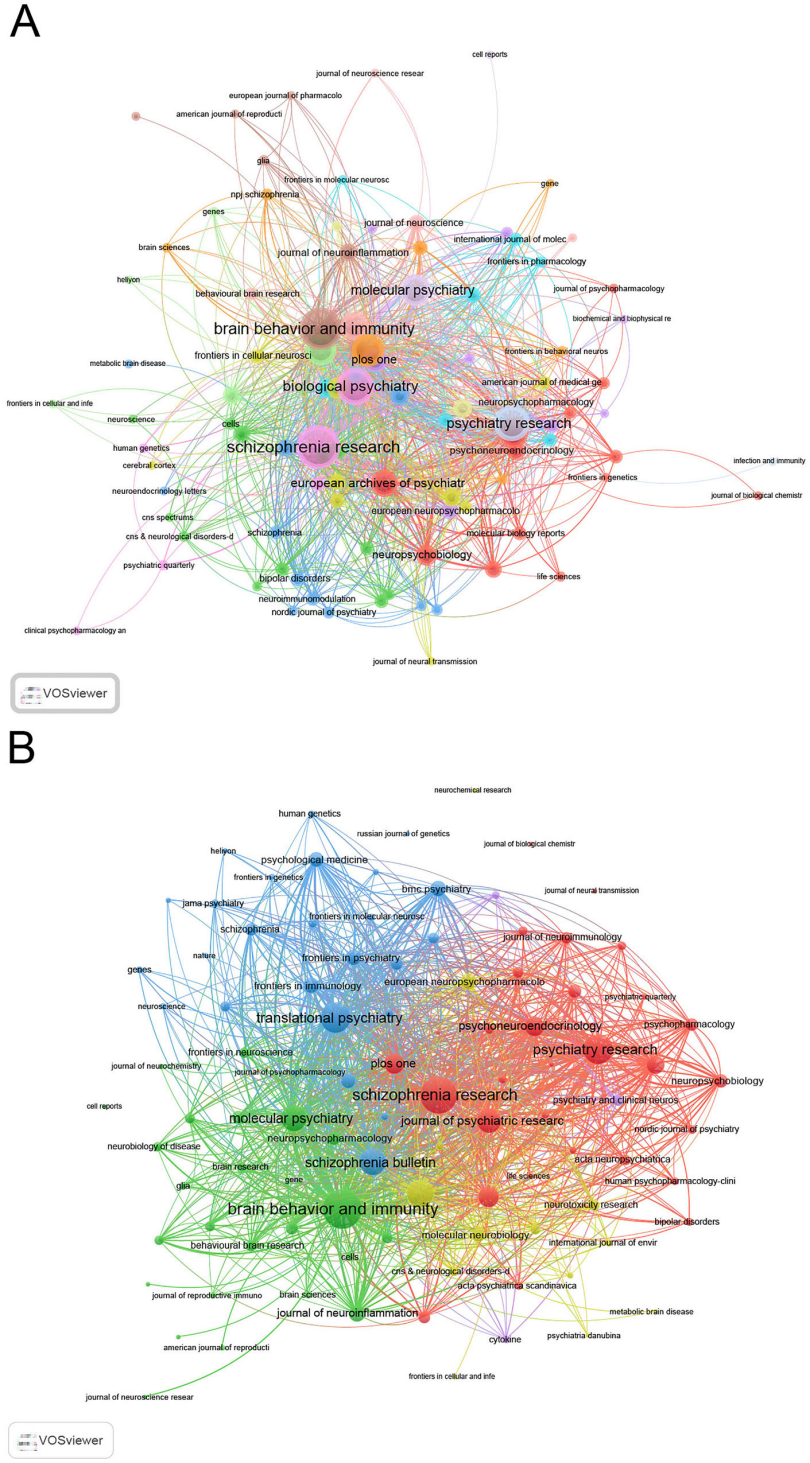
Analysis of journals publishing schizophrenia and immune response research. (A) Co‐occurrence networks of journals, showing how journals are interconnected through shared research themes. (B) Coupling networks of journals, illustrating how journals influence one another through shared citations and research focus.

The journal co‐occurrence network (Figure 6A) identified 98 journals with at least three occurrences. Co‐occurrence analysis highlights that the three journals with the highest total link strength in this network were *Schizophrenia Research* (731), *Brain, Behavior, and Immunity* (687), and *Biological Psychiatry* (516). The coupling network (Figure 6B) included 99 journals with at least three coupled occurrences, revealing the three key journals with the highest total link strength: *Brain, Behavior, and Immunity* (38,336), *Schizophrenia Research* (33,538), and *Translational Psychiatry* (22,798). These linkages suggest that these journals not only publish high‐impact research but also serve as key nodes for interdisciplinary knowledge exchange, fostering collaborations between psychiatric, immunological, and molecular research communities.

### 3.6 Analysis of Keyword Co‐Occurrence and Bursts

**FIGURE 7:**
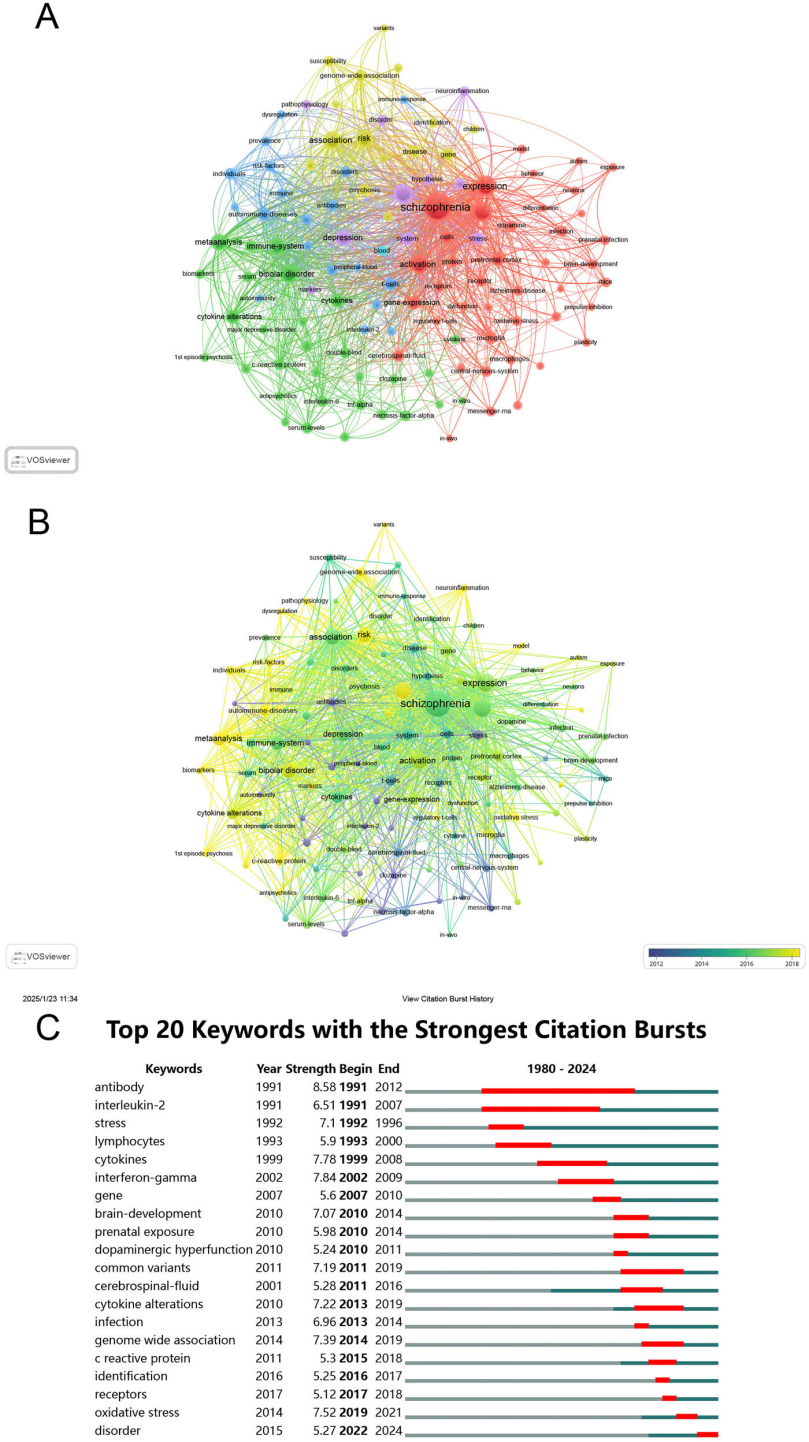
Analysis of keywords in schizophrenia and immune response research. (A) Visual analysis of the keyword co‐occurrence network, identifying major themes such as neuroinflammation and oxidative stress. (B) Time‐trend analysis of keyword co‐occurrence, illustrating shifts in research focus over time. (C) Top 20 keywords with the strongest citation bursts, highlighting emerging areas of interest such as precision psychiatry and immune biomarkers.

Keyword co‐occurrence analysis revealed five distinct research clusters (Figure 7A). **Cluster 1** (red, neuroinflammation and cellular mechanisms) focused on neuroinflammation and cellular mechanisms related to schizophrenia. Prominent keywords include “oxidative stress,” “microglia,” “dopamine,” “gene expression,” “immune activation,” and “neurons.” **Cluster 2** (green, immune markers and clinical responses) highlighted immune‐related biomarkers and clinical responses. Key terms include “cytokine alterations,” “CRP,” “inflammatory markers,” “antipsychotics,” “bipolar disorder,” and “interleukin‐6.” **Cluster 3** (blue, autoimmunity and peripheral immune system) centered on autoimmune mechanisms and peripheral immune system involvement in schizophrenia. Important keywords include “autoimmune diseases,” “antibodies,” “lymphocytes,” and “T cells.” **Cluster 4** (yellow, genetic susceptibility and pathways) explored genetic susceptibility and molecular pathways. Key terms include “genome‐wide association,” “polymorphisms,” “common variants,” “gene,” and “risk factors.” **Cluster 5** (purple, neuroinflammation and stress) focused on neuroinflammation and stress‐related mechanisms in schizophrenia. Key terms include “neuroinflammation,” “stress,” “inflammation,” “markers,” and “pathophysiology.”

The temporal evolution of research themes demonstrated the progression of research focus over time (Figure 7B). Early research focused on foundational immune markers like “cytokines” and “immune system.” More recent studies have concentrated on emerging keywords such as “meta‐analysis,” “genome‐wide association,” and “oxidative stress,” which are particularly prominent in the yellow‐coded nodes.

The analysis of keyword bursts further supports these findings, with 20 terms showing significant increases in citation frequency over specific periods (Figure 7C). Early bursts (1991–2000) were dominated by basic immunological terms, such as “antibody” (strength = 8.58) and “interleukin‐2” (strength = 6.51). The middle period (2001–2010) saw a shift towards terms related to biological mechanisms, including “interferon‐gamma” (strength = 7.84) and “gene” (strength = 5.6). Recent bursts (2011–2024), with the term “cytokine alterations,” demonstrated one of the most significant bursts (strength = 7.22) between 2013 and 2019. Other terms such as “infection” (strength = 6.96, 2013–2014) and “cerebrospinal fluid” (strength = 5.28, 2011–2016) reflect the ongoing exploration of immune‐related biomarkers and their role in disease progression. “Genome‐wide association” (strength = 7.39, 2014–2019) and “common variants” (strength = 7.19) experienced notable bursts from 2014 to 2019 and 2011 to 2019, respectively. The keyword “oxidative stress” showed strong citation activity (strength = 7.52) from 2019 to 2021. Notably, “disorder” exhibited a recent burst (strength = 5.27) from 2022 to 2024.

## 4 Discussion

This bibliometric analysis provides a comprehensive overview of global research trends on schizophrenia and immune response, revealing significant advances and persistent gaps in the field. Our findings highlight the shift towards integrating immune mechanisms into schizophrenia research, reflecting a broader transition from neurotransmitter‐based models to interdisciplinary frameworks that incorporate immunology, genetics, and environmental factors.

The dominance of countries like the United States, China, and Germany in publication output reflects their strong research infrastructure, substantial funding, and strategic national initiatives. For example, the US National Institute of Mental Health (NIMH) has prioritized neuroimmune mechanisms (National Academies of Sciences E, Medicine, Policy, Global A, Government‐University‐Industry Research R 2021), whereas China's “Healthy China 2030” initiative has fueled rapid progress in biomedical research (Sanislow et al. 2022; Kong et al. 2023). Germany's legacy of excellence in psychiatric genetics and immunology has also positioned it as a leader in the field. These trends underscore the role of targeted funding and policy frameworks in advancing research priorities, suggesting that similar investments in underrepresented regions could expand the global research landscape.

Institutions, such as the University of California System, Harvard University, and the University of Oslo, have emerged as key contributors due to their integration of interdisciplinary teams, access to large patient cohorts, and participation in international consortia (Schijven et al. 2023). Their success highlights the value of collaborative networks and resource sharing in driving high‐impact research, particularly in underexplored areas like immune‐based interventions and genetic research. Expanding these collaborations to regions with limited research infrastructure could address geographic disparities and foster innovation (Dwir et al. 2023).

The influence of journals, like *Brain, Behavior, and Immunity* and *Schizophrenia Research*, reflects their role in advancing interdisciplinary studies at the intersection of psychiatry and immunology (Dickerson et al. 2017). These journals promote rapid dissemination of novel findings, shaping the field's focus on neuroinflammation, immune markers, and genetic‐immune interactions (Harvey et al. 2019). Their high citation rates and IF indicate that research published in these venues not only advances scientific understanding but also informs clinical practice.

Leading authors, such as Maes M., Müller N., and Yolken R. H., have contributed foundational work on inflammation, cytokine imbalances, and infectious agents in schizophrenia (Solmi et al. 2023; Severance et al. 2016). Collaboration patterns show that international partnerships, especially among European and US researchers, enhance impact, whereas limited cross‐regional collaboration in some areas highlights a need for broader consortia and shared resources (Solmi et al. 2023).

### 4.1 Insights From Keyword Clusters

The keyword clusters identified—neuroinflammation and cellular mechanisms, immune markers and clinical responses, autoimmunity and peripheral immune system, genetic susceptibility and pathways, and neuroinflammation and stress—reflect the diversification and maturation of schizophrenia research. Early investigations (1991–2000) predominantly focused on basic immune markers, such as cytokines and antibodies, which established foundational links between schizophrenia and immune dysfunction (Noy et al. 1994; Moore et al. 1998). Over time, the field evolved to explore more sophisticated mechanisms involving oxidative stress, microglial activation, and systemic inflammation, which are now recognized as key contributors to synaptic deficits, cognitive impairments, and negative symptoms in schizophrenia (Luo et al. 2014; Cho et al. 2019).

The prominence of neuroinflammation and oxidative stress highlights their central roles in schizophrenia's pathophysiology. Oxidative stress, for example, has been implicated in neuronal damage and synaptic dysfunction, whereas microglial activation contributes to immune‐mediated synaptic pruning (Luo et al. 2014; Gaebel et al. 2016). These processes are particularly relevant to treatment‐resistant subgroups, emphasizing the need for further exploration of how neuroinflammation drives disease heterogeneity. Similarly, the identification of immune‐related biomarkers, such as elevated CRP and altered cytokine levels, underscores their potential utility in stratifying patients and guiding personalized treatments (Mokhtari and Lachman 2016; Miller et al. 2014; Bellaagh Johansson et al. 2025). However, many gaps remain, particularly in understanding the interplay between central and peripheral immune markers and their implications for early diagnosis and treatment outcomes.

Another emerging focus is the role of genetic susceptibility in schizophrenia, particularly immune‐related loci within the major histocompatibility complex (MHC). These loci are thought to influence both neurodevelopmental processes and immune function, providing a potential link between genetic predisposition and the immune dysregulation observed in schizophrenia (Harvey et al. 2019). Advances in genome‐wide association studies (GWAS) have the potential to integrate genetic data with clinical and immune phenotypes, paving the way for precision psychiatry approaches that target specific immune pathways.

Lastly, the interplay between stress and neuroinflammation represents an increasingly important area of research, as environmental stressors are known to activate inflammatory pathways, exacerbate oxidative stress, and worsen clinical outcomes in schizophrenia (Alotiby 2024; Ravi et al. 2021). Understanding how stress interacts with immune dysfunction may inform novel interventions aimed at mitigating these effects, particularly in high‐risk populations.

### 4.2 Precision Medicine and Future Directions

Although the term “precision medicine” frequently appears in the literature, its application in schizophrenia research remains in its early stages. Multi‐omics approaches, which integrate immune, genetic, and environmental data, offer promising tools for patient stratification and the development of personalized therapies. For example, elevated CRP levels have been linked to treatment resistance, suggesting that immune biomarkers could guide treatment decisions in specific subgroups (Miller et al. 2014). Similarly, immune‐related genetic loci identified through GWAS provide potential targets for immunomodulatory interventions (Harvey et al. 2019).

Looking ahead, future research should prioritize the integration of multi‐omics approaches to unravel the complex interplay between immune, genetic, and environmental factors in schizophrenia. Expanding international collaborations, particularly in underrepresented regions, could diversify perspectives and accelerate progress. Additionally, the development of immune‐based diagnostics and preventive interventions could improve early detection and patient outcomes, aligning with the goals of precision psychiatry (Ermakov et al. 2022; Yu et al. 2024).

### 4.3 Limitations

This study has several limitations. First, the reliance on a single database (WoSCC) and a focus on English‐language publications may have excluded valuable contributions from other databases and non‐English‐speaking regions. Second, the use of bibliometric tools may introduce biases related to database coverage and indexing methods. Future studies should employ multi‐database searches, incorporate multilingual literature, and refine analytical methods to enhance the comprehensiveness and reliability of findings. Despite these limitations, this study provides a robust foundation for understanding global trends and identifying future research priorities in schizophrenia and immune response.

## 5 Conclusion

This bibliometric analysis elucidates the dynamic evolution of research on schizophrenia and immune response. Keyword analysis indicates that mechanistic studies, particularly those focusing on the immune system's role, remain a central focus. Future research could integrate clinical phenotypes with molecular mechanisms, leveraging multi‐omics data to deeply explore the role of immune regulation in the pathogenesis, progression, and treatment of schizophrenia. Furthermore, interdisciplinary collaboration and big data analytics hold promise for identifying novel biomarkers, thereby providing a theoretical foundation and practical guidance for advancing precision medicine.

## Author Contributions

Yu Zhang carried out the studies, participated in collecting data, drafted the manuscript, performed the statistical analysis, and participated in its design and drafted the manuscript.

## Funding

The author has nothing to report.

## Ethics Statement

The author has nothing to report.

## Conflicts of Interest

The author declares no conflicts of interest.

## Supporting information



Table S1 Publication and citation profiles of leading countries.
Table S2 Publication and citation profiles of high‐impact authors.
Table S3 Bibliometric indicators of high‐impact journals.

## Data Availability

All data generated or analyzed during this study are included in this published article.
