# Supplementary material for: Research Hotspots and Emerging Trends of Schizophrenia and Immune Response: A Bibliometric Analysis
Source: Brain Behav. 2025 Nov 21;15(11):e71054. doi: 10.1002/brb3.71054 (PMC12638435; doi:10.1002/brb3.71054)
Supplement: Supplementary file 1 — Table S1 Publication and citation profiles of leading countries. Table S2 Publication and citation profiles of high‐impact authors. Table S3 Bibliometric indicators of high‐impact journals. [file BRB3-15-e71054-s001.docx]

**Table S1 Publication and Citation Profiles of Leading Countries**

| **Country** | **Articles** | **Freq** | **SCP** | **MCP** | **MCP-Ratio** | **TP** | **TP-rank** | **TC** | **TC-rank** | **Average Citations** |
| --- | --- | --- | --- | --- | --- | --- | --- | --- | --- | --- |
| USA | 364 | 0.234 | 254 | 110 | 0.302 | 1886 | 1 | 18328 | 1 | 50.4 |
| CHINA | 208 | 0.134 | 136 | 72 | 0.346 | 925 | 2 | 3897 | 4 | 18.7 |
| GERMANY | 112 | 0.072 | 70 | 42 | 0.375 | 520 | 3 | 5706 | 3 | 50.9 |
| UK | 77 | 0.049 | 29 | 48 | 0.623 | 408 | 4 | 8572 | 2 | 111.3 |
| JAPAN | 61 | 0.039 | 47 | 14 | 0.23 | 226 | 10 | 2250 | 5 | 36.9 |
| AUSTRALIA | 58 | 0.037 | 20 | 38 | 0.655 | 339 | 5 | 2008 | 7 | 34.6 |
| ITALY | 48 | 0.031 | 32 | 16 | 0.333 | 226 | 9 | 1786 | 9 | 37.2 |
| BRAZIL | 39 | 0.025 | 15 | 24 | 0.615 | 205 | 11 | 1152 | 13 | 29.5 |
| ISRAEL | 39 | 0.025 | 33 | 6 | 0.154 | 157 | 14 | 1923 | 8 | 49.3 |
| SPAIN | 38 | 0.024 | 27 | 11 | 0.289 | 287 | 6 | 1247 | 10 | 32.8 |
| POLAND | 37 | 0.024 | 28 | 9 | 0.243 | 139 | 17 | 867 | 15 | 23.4 |
| NETHERLANDS | 35 | 0.022 | 21 | 14 | 0.4 | 242 | 8 | 2035 | 6 | 58.1 |
| CANADA | 34 | 0.022 | 22 | 12 | 0.353 | 196 | 12 | 1001 | 14 | 29.4 |
| NORWAY | 28 | 0.018 | 12 | 16 | 0.571 | 285 | 7 | 327 | 25 | 11.7 |
| SWEDEN | 28 | 0.018 | 15 | 13 | 0.464 | 146 | 15 | 1206 | 11 | 43.1 |
| FRANCE | 25 | 0.016 | 13 | 12 | 0.48 | 166 | 13 | 474 | 22 | 19 |
| TURKEY | 25 | 0.016 | 24 | 1 | 0.04 | 79 | 22 | 571 | 17 | 22.8 |
| DENMARK | 24 | 0.015 | 10 | 14 | 0.583 | 141 | 16 | 821 | 16 | 34.2 |
| KOREA | 24 | 0.015 | 18 | 6 | 0.25 | 91 | 19 | 1158 | 12 | 48.2 |
| RUSSIA | 24 | 0.015 | 22 | 2 | 0.083 | 84 | 21 | 311 | 26 | 13 |

Note(s): Articles: Publications of Corresponding Authors only. Freq: Frequence of Total Publications. MCP-Ratio: Proportion of Multiple Country Publications. TP: Total Publications. TP-rank: Rank of Total Publications. TC: Total Citations. TC-rank: Rank of Total Citations. Average Citations: The average number of citations

**Table S2 Publication and Citation Profiles of High-Impact Authors**

| **Author** | **h-index** | **g-index** | **m-index** | **PY-start** | **TP** | **TP-Frac** | **TP-rank** | **TC** | **TC-rank** |
| --- | --- | --- | --- | --- | --- | --- | --- | --- | --- |
| MAES M | 21 | 35 | 0.677 | 1995 | 35 | 7.58 | 1 | 2114 | 4 |
| MÜLLER N | 19 | 21 | 0.704 | 1999 | 21 | 6.11 | 5 | 1788 | 5 |
| YOLKEN RH | 19 | 27 | 1.188 | 2010 | 27 | 4.12 | 2 | 1227 | 7 |
| YOLKEN R | 16 | 23 | 0.842 | 2007 | 23 | 2.94 | 4 | 1195 | 8 |
| WEICKERT CS | 15 | 21 | 1.154 | 2013 | 21 | 2.53 | 5 | 1236 | 6 |
| BAHN S | 14 | 18 | 0.737 | 2007 | 18 | 2.06 | 9 | 958 | 13 |
| DICKERSON F | 14 | 19 | 0.737 | 2007 | 19 | 2.94 | 8 | 1178 | 9 |
| DREXHAGE HA | 14 | 18 | 0.538 | 2000 | 18 | 2.58 | 9 | 1020 | 10 |
| STEINER J | 14 | 17 | 0.7 | 2006 | 17 | 2.01 | 11 | 988 | 12 |
| ANDREASSEN OA | 13 | 25 | 1 | 2013 | 25 | 1.73 | 3 | 6071 | 2 |
| KAHN RS | 13 | 16 | 0.929 | 2012 | 16 | 1.92 | 12 | 6584 | 1 |
| RIEDEL M | 13 | 15 | 0.481 | 1999 | 15 | 2.31 | 13 | 1001 | 11 |
| BOGERTS B | 12 | 12 | 0.6 | 2006 | 12 | 1.41 | 19 | 790 | 17 |
| KHUSHALANI S | 12 | 12 | 0.75 | 2010 | 12 | 1.45 | 19 | 822 | 14 |
| ORIGONI A | 12 | 13 | 0.632 | 2007 | 13 | 1.8 | 14 | 800 | 15 |
| STALLINGS C | 12 | 13 | 0.632 | 2007 | 13 | 1.8 | 14 | 800 | 15 |
| AROLT V | 11 | 13 | 0.367 | 1996 | 13 | 1.9 | 14 | 625 | 18 |
| DJUROVIC S | 11 | 20 | 0.846 | 2013 | 20 | 1.26 | 7 | 5986 | 3 |
| SEVERANCE EG | 11 | 13 | 0.688 | 2010 | 13 | 1.96 | 14 | 606 | 19 |
| TEIXEIRA AL | 11 | 13 | 0.786 | 2012 | 13 | 1.42 | 14 | 434 | 20 |

Note(s): H-index: The h-index of the journal, which measures both the productivity and citation impact of the publications. G-index: The G-index of the journal, which gives more weight to highly-cited articles. M-index: The m-index of the journal, which is the h-index divided by the number of years since the first published paper. TP: Total Publications. TP-rank: Rank of Total Publications. TC: Total Citations. TC-rank: Rank of Total Citations. Average Citations: The average number of citations per publication. PY-start: Publication Year Start, indicating the year the journal started publication.

**Table S3 Bibliometric Indicators of High-Impact Journals**

| **Journal** | **H-index** | **G-index** | **M-index** | **IF** | **JCR** | **TP** | **TP-rank** | **TC** | **TC-rank** | **PY-start** |
| --- | --- | --- | --- | --- | --- | --- | --- | --- | --- | --- |
| BRAIN BEHAVIOR AND IMMUNITY | 37 | 66 | 1.423 | 8.8 | 1 | 96 | 1 | 1877 | 4 | 2000 |
| SCHIZOPHRENIA RESEARCH | 36 | 59 | 1.029 | 3.6 | 1 | 84 | 2 | 3253 | 1 | 1991 |
| SCHIZOPHRENIA BULLETIN | 25 | 38 | 0.543 | 5.3 | 1 | 38 | 6 | 1832 | 5 | 1980 |
| TRANSLATIONAL PSYCHIATRY | 24 | 46 | 1.846 | 5.8 | 1 | 56 | 4 | 904 | 16 | 2013 |
| BIOLOGICAL PSYCHIATRY | 23 | 27 | 0.523 | 9.6 | 1 | 27 | 10 | 2991 | 2 | 1982 |
| MOLECULAR PSYCHIATRY | 23 | 37 | 0.852 | 9.6 | 1 | 37 | 7 | 2381 | 3 | 1999 |
| PSYCHIATRY RESEARCH | 23 | 38 | 0.657 | 4.2 | 1 | 56 | 3 | 1136 | 12 | 1991 |
| PLOS ONE | 22 | 36 | 1.158 | 2.9 | 1 | 42 | 5 | 1245 | 9 | 2007 |
| JOURNAL OF PSYCHIATRIC RESEARCH | 18 | 36 | 0.667 | 3.7 | 1 | 36 | 8 | 795 | 19 | 1999 |
| PROGRESS IN NEURO-PSYCHOPHARMACOLOGY & BIOLOGICAL PSYCHIATRY | 17 | 32 | 0.944 | 5.3 | 1 | 32 | 9 | 1092 | 13 | 2008 |
| EUROPEAN ARCHIVES OF PSYCHIATRY AND CLINICAL NEUROSCIENCE | 13 | 18 | 0.394 | 3.5 | 1 | 18 | 14 | 549 | 28 | 1993 |
| PSYCHONEUROENDOCRINOLOGY | 13 | 19 | 0.464 | 3.4 | 2 | 19 | 13 | 437 | 39 | 1998 |
| JOURNAL OF NEUROIMMUNOLOGY | 11 | 17 | 0.344 | 2.9 | 2 | 17 | 17 | 549 | 29 | 1994 |
| JOURNAL OF NEUROINFLAMMATION | 11 | 16 | 0.786 | 9.3 | 1 | 16 | 19 | 429 | 40 | 2012 |
| MEDICAL HYPOTHESES | 11 | 18 | 0.314 | 2.1 | 3 | 18 | 15 | 269 | 62 | 1991 |
| SCIENTIFIC REPORTS | 10 | 20 | 0.909 | 3.8 | 1 | 24 | 11 | 456 | 37 | 2015 |
| EUROPEAN NEUROPSYCHOPHARMACOLOGY | 9 | 13 | 0.409 | 6.1 | 1 | 13 | 22 | 273 | 61 | 2004 |
| JOURNAL OF AFFECTIVE DISORDERS | 9 | 16 | 0.281 | 4.9 | 1 | 16 | 18 | 606 | 23 | 1994 |
| NEUROPSYCHOBIOLOGY | 9 | 14 | 0.205 | 2.3 | 2 | 14 | 21 | 256 | 65 | 1982 |
| NEUROPSYCHOPHARMACOLOGY | 9 | 11 | 0.375 | 6.6 | 1 | 11 | 27 | 3 | 2178 | 2002 |

Note(s): H-index: The h-index of the journal, which measures both the productivity and citation impact of the publications. IF: Impact Factor, indicating the average number of citations to recent articles published in the journal. JCR-Quartile: The quartile ranking of the journal in the Journal Citation Reports, indicating the journal's ranking relative to others in the same field (Q1: top 25%, Q2: 25%-50%, Q3: 50%-75%, Q4: bottom 25%). TP: Total Publications. TP-rank: Rank of Total Publications. TC: Total Citations. TC-rank: Rank of Total Citations. Average Citations: The average number of citations per publication. PY-start: Publication Year Start, indicating the year the journal started publication.
